# Identification of 2 Novel Subtypes of Hepatitis C Virus Genotype 8 and a Potential New Genotype Successfully Treated With Direct Acting Antivirals

**DOI:** 10.1093/infdis/jiae253

**Published:** 2024-05-08

**Authors:** Jean L Mbisa, Zena Lapp, David F Bibby, Laura T Phillips, Carmen F Manso, Simon Packer, Ruth Simmons, Kathryn Harris, Jaiganesh Mohan, Lalitha Chinnappan, Thomas Leitner, Daniel Bradshaw

**Affiliations:** Virus Reference Department, UK Health Security Agency, London, United Kingdom; National Institute for Health and Care Research Health Protection Research Unit (NIHR HPRU) in Bloodborne and Sexually Transmitted Infections, London, United Kingdom; Theoretical Biology and Biophysics Group, Los Alamos National Laboratory, Los Alamos, New Mexico, USA; Virus Reference Department, UK Health Security Agency, London, United Kingdom; Virus Reference Department, UK Health Security Agency, London, United Kingdom; National Institute for Health and Care Research Health Protection Research Unit (NIHR HPRU) in Bloodborne and Sexually Transmitted Infections, London, United Kingdom; Virus Reference Department, UK Health Security Agency, London, United Kingdom; Virus Reference Department, UK Health Security Agency, London, United Kingdom; Virus Reference Department, UK Health Security Agency, London, United Kingdom; National Institute for Health and Care Research Health Protection Research Unit (NIHR HPRU) in Bloodborne and Sexually Transmitted Infections, London, United Kingdom; Royal London Hospital, Barts Health NHS Trust, London, United Kingdom; Warrington and Halton Teaching Hospitals NHS Foundation Trust, Warrington, United Kingdom; Warrington and Halton Teaching Hospitals NHS Foundation Trust, Warrington, United Kingdom; Theoretical Biology and Biophysics Group, Los Alamos National Laboratory, Los Alamos, New Mexico, USA; Virus Reference Department, UK Health Security Agency, London, United Kingdom; National Institute for Health and Care Research Health Protection Research Unit (NIHR HPRU) in Bloodborne and Sexually Transmitted Infections, London, United Kingdom

**Keywords:** HCV, whole-genome sequencing, genotype classification, DAA therapy, genetic diversity

## Abstract

**Background:**

Hepatitis C virus (HCV) has high genetic diversity and is classified into 8 genotypes and >90 subtypes, with some endemic to specific world regions. This could compromise direct-acting antiviral efficacy and global HCV elimination.

**Methods:**

We characterized HCV subtypes “rare” in the United Kingdom (non-1a/1b/2b/3a/4d) by means of whole-genome sequencing via a national surveillance program. Genetic analyses to determine the genotype of samples with unresolved genotypes were undertaken by comparison with International Committee on Taxonomy of Viruses HCV reference sequences.

**Results:**

Two HCV variants were characterized as being closely related to the recently identified genotype (GT) 8, with >85% pairwise genetic distance similarity to GT8 sequences and within the typical intersubtype genetic distance range. The individuals infected by the variants were UK residents originally from Pakistan and India. In contrast, a third variant was only confidently identified to be more similar to GT6 compared with other genotypes across 6% of the genome and was isolated from a UK resident originally from Guyana. All 3 were cured with pangenotypic direct-acting antivirals (sofosbuvir-velpatasvir or glecaprevir-pibrentasvir) despite the presence of resistance polymorphisms in NS3 (80K/168E), NS5A (28V/30S/62L/92S/93S) and NS5B (159F).

**Conclusions:**

This study expands our knowledge of HCV diversity by identifying 2 new GT8 subtypes and potentially a new genotype.

## Teaser

Two new hepatitis C virus (HCV) subtypes belonging to genotype 8 and a potentially new genotype may be circulating in low- and middle-income countries. More genomic surveillance and treatment outcome data in these countries are required to support HCV elimination.

Hepatitis C virus (HCV) is a positive-sense single-strand RNA virus belonging to the genus *Hepacivirus* and the family Flaviviridae. It is an important global human pathogen affecting an estimated 58 million individuals. It was responsible for approximately 290 000 deaths in 2019 because of end-stage liver disease caused by cirrhosis and cancer [1]. An estimated 1.5 million new HCV infections occur annually worldwide, and injecting drug use and unsafe healthcare practices are major risk factors [1]. In 2016, the World Health Assembly adopted the Global Health Sector Strategy on viral hepatitis, which advocates for the elimination of HCV as a public health threat by 2030 [2]. This is defined as an 80% reduction in HCV incidence and 65% reduction in deaths caused by HCV, using 2015 figures as a baseline.

In the United Kingdom, the number of individuals living with chronic HCV infection significantly declined to 92 900 in 2021, a 47.2% reduction compared with 2015 figures [3]. This has been facilitated by the high cure rate (>95%) of HCV direct-acting antivirals (DAAs), which have underpinned national treatment programs since 2015. However, most clinical trials to test the efficacy of DAAs have been carried out in the Western world where epidemic subtypes such as 1a, 1b, and 3a predominate. This raises concerns for reduced effectiveness against endemic subtypes that are more prevalent in low- and middle-income countries. Studies in recent years support these concerns and show that some endemic HCV subtypes are inherently resistant to DAAs [4]. These subtypes exhibit significantly lower (50%–90%) rates of sustained virological response at 12 weeks (SVR12) after completion of DAA therapy, including pangenotypic third-generation DAAs such as sofosbuvir-velpatasvir and glecaprevir-pibrentasvir [5–10]. Therefore, continued surveillance of HCV diversity and research into responses to DAA therapy will be important for achieving global HCV elimination.

Our knowledge of the diversity of HCV has expanded in the past decade with the advent of new sequencing technologies; the number of genotypes has increased from 6 to 8, and the number of subtypes from 67 to 93 [11–15]. HCV strains belonging to different genotypes differ at approximately 30%–35% of nucleotide positions, whereas strains that are different subtypes belonging to the same genotype differ at 15%–25% of nucleotide positions. The global distribution of HCV genotypes and subtypes also varies by geographic region, ethnicity and risk groups. Genotype (GT) 2 is endemic in West Africa, GT4 in Central and Eastern sub-Saharan Africa and the Middle East, GT5 in South Africa, and GT6 in Southeast Asia as well as migrant populations from these regions residing in the Western world [16]. The newly discovered GT7 and GT8 appear to be even more restricted geographically, with the former reported in a small number of individuals from the Democratic Republic of Congo and Uganda, and the latter was reported in patients originally from the Punjab region in India but now residing in Canada or Australia [12, 13, 17].

The implementation of a genotype-agnostic HCV whole-genome sequencing (WGS) service in the clinical pathway and for public health surveillance in the United Kingdom provides an opportunity to understand the diversity of HCV within the UK population [18]. Between August 2019 and April 2022, the service generated HCV WGS from 2340 people with diagnosed HCV, of whom 122 (5.2%) were infected with subtypes “rare” to the United Kingdom (non-1a, 1b, 2b, 3am and 4d). Here, we describe and characterize 3 new HCV variants, 1 that may belong to a new genotype and 2 that may belong to new subtypes in the recently identified GT8. We also show that all 3 strains responded successfully to pangenotypic DAA therapy.

## METHODS

### Patient Samples, Data and Ethics

The Antiviral Unit at the UK Health Security Agency (UKHSA) is a national reference laboratory that provides a UK Accreditation Service–approved HCV WGS service for genotyping and antiviral resistance testing for National Health Service (NHS) patients to inform direct clinical care. As part of surveillance to monitor the progress of national HCV elimination programs, viruses that are difficult to subtype using routine genotyping methods or considered “rare” subtypes in the United Kingdom (ie, not 1a, 1b, 2b, 3a, and 4d) are identified. The sequence data are linked to the HCV Treatment Registry, which collects clinical, treatment outcome, and epidemiological data from the NHS England Operational Delivery Networks that administer HCV treatment. UKHSA has approval under section 251 of the UK NHS Act 2006 and regulation 3 of the associated Health Service (Control of Patient Information) Regulations 2002 to collect patient-level data for public health monitoring purposes without patient consent, including for the current surveillance study (reference no. NIS_CRP_58_2020). This is reviewed annually by the UKHSA Caldicott Panel to ensure compliance with information governance policies. UKHSA's Research Ethics and Governance Group also approved the study.

### HCV WGS

The complete genomic sequencing of HCV was performed using a state-of-the-art sequence capture method, described elsewhere [18]. Briefly, the TAKARA SMARTer Stranded Total RNA-Seq Kit v2 was used to generate libraries from DNAse-digested nucleic acid extracts. Libraries were pooled according to both total mass and HCV-specific fragment frequency. Pools were hybridized to customized HCV-specific biotinylated oligonucleotide probes, designed to cover the diversity of HCV GT1-8, with enriched fragments partitioned onto streptavidin beads and subjected to further cycles of polymerase chain reaction (PCR) amplification before being sequenced on an Illumina MiSeq instrument. Bioinformatic analyses were performed as described elsewhere [18, 19]. Consensus sequences were submitted to GenBank, which includes predicted full-length polyproteins and partial 5′ and 3′ untranslated regions with mixed bases called at 15% variant frequency at a minimum read depth of 30 (accession nos. PP092205–PP092208).

### Phylogenetic Analyses and Pairwise Genetic Distance Calculations

We downloaded 238 HCV reference sequences from the International Committee on Taxonomy of Viruses (ICTV; version 09.03.22 and created an alignment with the 3 sequences from individuals with difficult-to-subtype viruses, using MAFFT software (FFT-NS-I; version 7.520) [20]. A maximum-likelihood phylogenetic tree was reconstructed with PhyML software (version 3.3) using the GTR + I + G substitution model, Nearest-Neighbour-Interchange (NNI) and Subtree-Pruning-and-Regrafting (SPR) for tree searching, and the approximate likelihood-ratio test to assess branch support [21, 22]. R software, version 4.3.1, was used for all analyses. Pairwise genetic (ie, Hamming) distances were computed for each pair of sequences using the ape::dist.dna function (version 5.7.1) with pairwise gap stripping [23]. Distances were computed across the whole genome and for sliding windows (size, 500 base pairs [bp]; step size, 50 bp).

Reference sequence pairs were categorized as between genotype, between subtype (within the same genotype), or within subtype. Within-genotype pairs with an unknown subtype were excluded from analyses. For each window and reference sequence pair category, we computed empirical 95% confidence intervals on the genetic distances by taking the 2.5% and 97.5% quantiles of the respective genetic distance distributions. For each patient's virus sequence, we compared the genetic distances between the sequence and the most closely related genotype (ie, the genotype of the reference sequence with the smallest genetic distance to the patient's virus sequence) with the genetic distances between the patient's virus sequence and each of the other genotypes, using a 1-sided Wilcoxon rank-sum test.

For each window in a patient's virus sequence, we calculated the genetic distance to each of the reference sequences of each genotype. We then compared the distribution of the genetic distances to the most closely related genotype versus the distribution of the distances to all other genotypes. From this we calculated the proportion of overlap between these 2 distributions using the overlapping::overlap function (version 2.1; type “2”) [24]. To evaluate whether this overlap indicated a significant assignment to the most closely related genotype, we compared it to the overlap of between-subtype versus between-genotype genetic distance distributions of established reference sequences (reference overlap). We considered the patient's virus sequence to be more closely related to the nearest genotype relative to other genotypes if the proportion of overlap between the distributions was less than the reference overlap. Otherwise, we considered it to be uncertain.

We also performed identical analyses, except (1) including the Pt2 sequence as an additional GT8 reference sequence and (2) with a representative reference sequence from each subtype, where we removed all reference strains of the same genotype or of the same subtype to investigate the signal observed when using a sequence with a known genotype and subtype. Code and data to recreate the genetic analyses and figures can be found at https://github.com/MolEvolEpid/novel_hcv_subtypes. We tested for signatures of recombination using SimPlot++, the PHI test, and PhyloPlace software with the branching index analysis method [25–27].

### Genotypic Resistance Analysis

Consensus sequences were submitted to Geno2pheno[hcv] and HCV-GLUE software for subtype attribution and/or resistance interpretation. This was supplemented with data from Sorbo et al [28], with any mutation that is associated with resistance in an epidemic subtype (1a, 1b, 2b, 3a, or 4d), defined as a resistance-associated substitution (RAS) and any other mutation that is not wild type in epidemic subtypes considered possible RASs.

## RESULTS

We describe 3 novel HCV variants identified in samples referred to the UK national reference laboratory due to indeterminate or unusual genotype results obtained using routine molecular methods (Table 1). The viruses were identified as belonging to subtype 5a, untyped, or GT1 of unspecified subtype using routine genotyping assays. HCV-GLUE software [29] identified all 3 consensus WGS sequences (for Pt1, Pt2, and Pt3) as HCV of unknown genotype whereas Geno2pheno[hcv] software [30] identified them as different subtypes belonging to GT4, GT5, or GT6 in the NS3, NS5,A and NS5B gene regions, with similarities to the closest reference sequences ranging from 69.7% to 76.5% (Table 1). Homology comparisons showed that the closest sequence identities were subtype 8a for Pt1 and Pt2 at 74.4% and 81.1%, respectively, and subtype 6j for Pt3 at 71.2%.

**Table 1.** Characteristics of Patients and Novel Variants

Patient (Sex)	Country of Birth	Ethnicity	Initial Genotype (Assay Type)^a^	Genotype by HCV-GLUE	Genotype by Geno2pheno[hcv]^b^			Genotype by Homology^c^
					NS3	NS5A	NS5B	
Pt1 (M)	India (South)	Asian	5a (LiPA)	HCV (unknown subtype)	6w (74.2%; DQ278892	5a (70.8%; AF064490)	6xa (73.4%; EU408330	8a (74.5%; MH590701)
Pt2 (F)	Pakistan	Asian	Untyped (NS5B sequencing)	HCV (unknown subtype)	6xb (71.1%; JX183552)	4d (69.7%; DQ418786)	4q (73.0%; FJ462434)	8a (81.5%; MH590701)
Pt3 (F)	Guyana	Indo-Asian	1—unspecified subtype (NS5B sequencing)	HCV (unknown subtype)	6r (75.3%; EU408328)	6*12 (72.4%; KJ470623)	6a (76.5%; Y12083)	6j (71.2%; KM587630)

Abbreviations: F, female; HCV, hepatitis C virus; M, male; Pt, patient.

^a^Performed by local laboratory.

^b^Geno2pheno[hcv] software, version 0.92 (last updated June 2019; accession nos. of reference sequences used are given for each subtype).

^c^Using the complete coding region of the patient's virus genome with reference HCV subtypes curated by the International Committee on Taxonomy of Viruses. The percentage in brackets represents the similarity to the closest reference with Accession no. provided.

**Figure 1.**
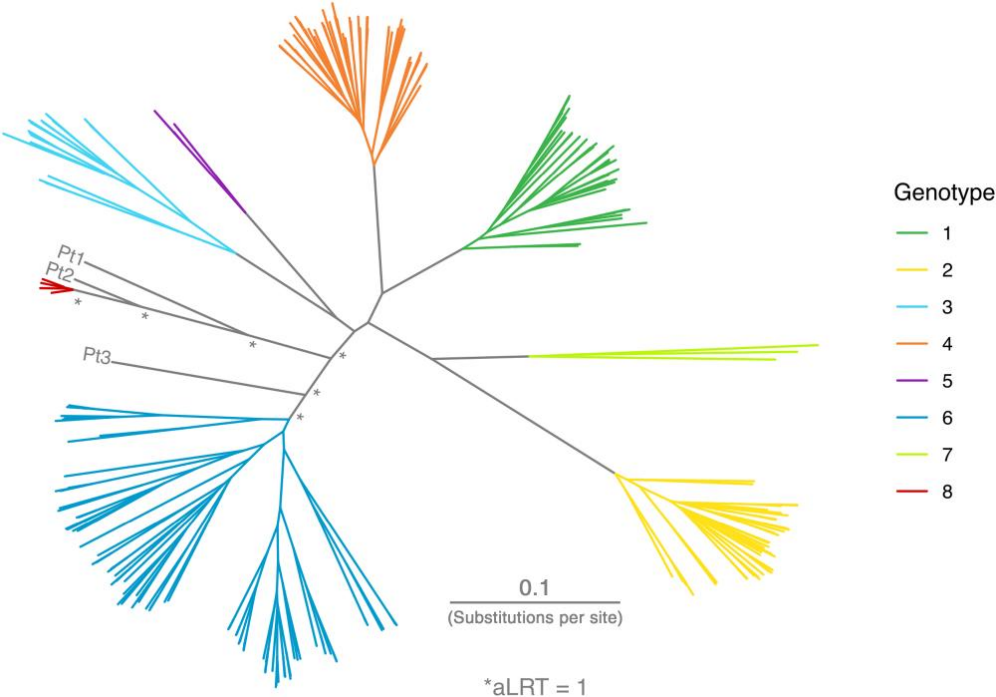
Maximum likelihood phylogeny of hepatitis C virus reference genotypes and 3 novel variant sequences. Reference sequences are colored by genotype, and the novel variants are labeled. Approximate likelihood-ratio test (aLRT) values are equal to 1 at all branches of interest (*asterisks*). Abbreviation: Pt, patient.

**Figure 2.**
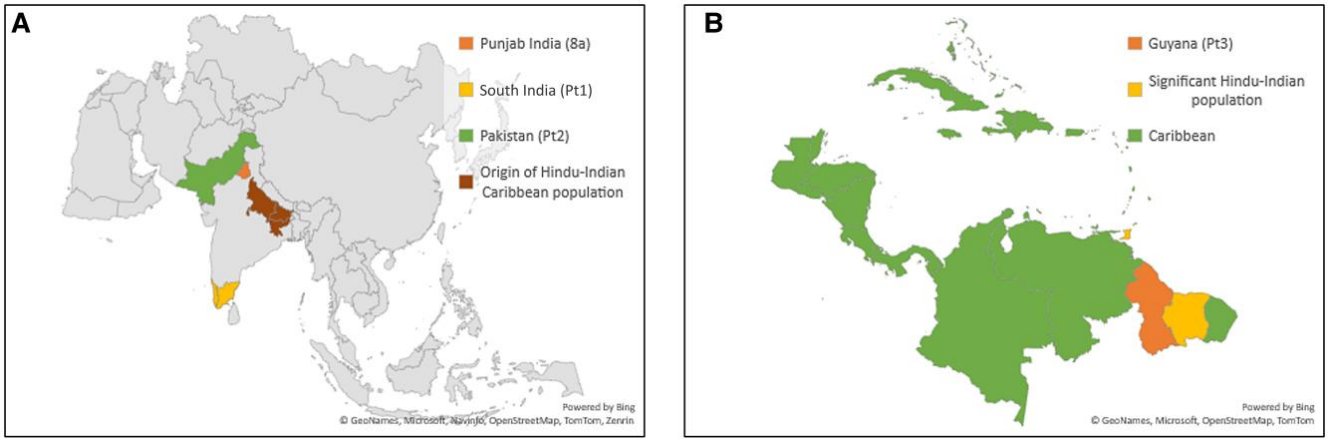
Potential geographic distribution of novel variants. *A*, Map of the subcontinent of India showing the regions where individuals infected with the novel variants may have originated from, compared with those infected with subtype 8a. For patient (Pt) 3, this shows the region where most Indian migrants to Guyana originated from. *B*, Map of the Caribbean showing Guyana, where the individual infected with a potential novel genotype (Pt3) originated from and the 2 other countries (Suriname and Trinidad and Tobago) with high Hindu-Indian populations.

Phylogenetic reconstruction confirmed the close clustering of Pt1 and Pt2 sequences to GT8 with high branch support (approximate likelihood-ratio test value, 1; Figure 1). These sequences were from individuals of Asian ethnicity who were born in India and Pakistan, respectively, but now reside in the United Kingdom (Table 1 and Figure 2*A*). On the other hand, Pt3 sequence was located between GT6 and GT8. This sequence was isolated from an individual of Asian ethnicity and Indian ancestry who was born in Guyana but now resides in the United Kingdom (Table 1 and Figure 2*B*). Genomes derived from both sequence capture and metagenomic data sets for the Pt1 virus were missing approximately 1155 bp spanning the p7 to NS2 gene region. The gap was filled from amplicons generated using target-specific PCR primers designed against the known flanking regions.

**Figure 3.**
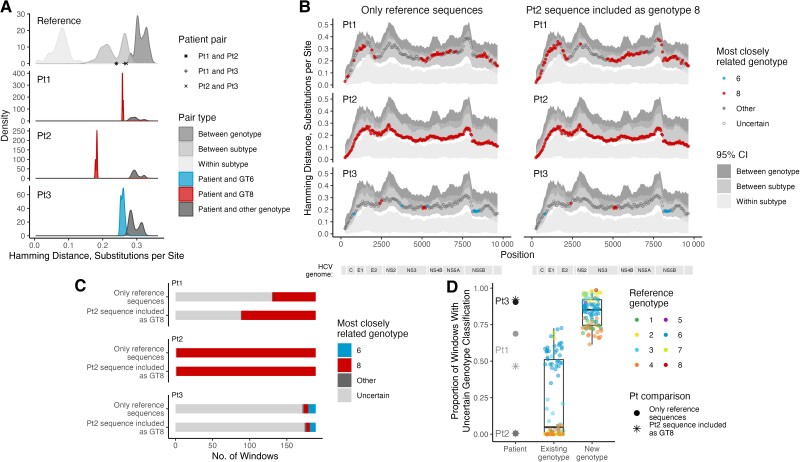
Pairwise genetic distance comparisons. *A*, Pairwise genetic distance distributions across the entire genome for reference-reference sequence pairs (*top panel*) and novel variant-reference sequence pairs (*bottom 3 panels*). For the novel variant panels, the pairwise distance distribution of the nearest genotype is presented in color. Points on the x-axis of the top panel represent pairwise distances between the novel variant pairs. *B*, Minimum pairwise genetic distance between a given novel variant and all reference sequences, using a sliding window size of 500 base pairs (bp) with a step size of 50 bp. Each point is colored by the most closely related genotype if that genotype is more closely related to the novel variant sequence than other genotypes; otherwise, the point is depicted as an open gray circle (see Methods and Supplementary Figure 1 for more details). Gray background indicates the 95% confidence interval (CI) for pairwise distances of each reference pair category type. Hepatitis C virus (HCV) genes are depicted below the x-axis. Plots on the left are comparisons with International Committee on Taxonomy of Viruses (ICTV) reference sequences only; plots on the right, with the Pt2 sequence included as a genotype (GT) 8 reference. *C*, Genotype uncertainty of novel variant sequences. The number of sliding windows for each novel variant sequence that were considered more closely related to a certain genotype or of uncertain genotype were quantified using ICTV reference sequences or when the Pt2 sequence was included as a GT8 reference 8. *D*, Comparison of genotype uncertainty in each novel variant with uncertainty observed using known subtypes, assuming that it does not belong to a known genotype or subtype by removing reference sequences of the same subtype (existing genotype) or of the same genotype (new genotype) from the comparative reference data set.

We further computed the pairwise genetic distances of the 3 novel variant genomes against ICTV reference sequences for GT1–G8. This showed that Pt1 and Pt2 sequences were more closely related to GT8 than to other genotypes (*P* < .05; Wilcoxon) but that their genetic distances fell within the intersubtype range even when they were compared against each other, suggesting that they were 2 new different subtypes within GT8 (Figure 3*A*). In contrast, Pt3 was closely related to GT6 (*P* < .05). However, the distribution of pairwise genetic distances for Pt3 sequence and GT6 sequences slightly overlaps that for Pt3 sequence and other genotypes exclusive of GT6 sequences (Figure 3*A*).

Further analysis of the similarity of Pt2 sequence to GT1–G8 using sliding windows across the genome confirmed that it was more closely related to GT8 than any other genotype across 99.5% (188 of 189) of the sliding windows, with the genetic distances falling within the 95% confidence interval for intersubtype genetic distance range in 88.9% (168 of 189) of the windows (Figure 3*B* and 3*C* and Supplementary Figure 1). In contrast, for some parts of the Pt1 and Pt3 genomes there was more uncertainty as to which genotype was most closely related (Figure 3*B* and 3*C* and Supplementary Figure 1); Pt1 and Pt3 did not exhibit evidence of being recombinants using the PHI test (*p* = 1), SimPlot++, or PhyloPlace software. When Pt2 was included in the reference data set as GT8, the percentage of Pt1 that was robustly identified as being similar to GT8 increased from 30.7% (58 of 189) to 52.9% (100 of 189), but this had no effect on Pt3, which was similar to GT6 in <6% of sliding windows under both scenarios (Figure 3*B* and 3*C* and Supplementary Figure 1). To further assess the classifications, we compared the proportion of windows with uncertain genotype classification to the expected distributions where genotypes preexisted (“existing genotype”) or not (“new genotype”). This revealed that Pt3 was best described by the “new genotype” distribution (interquartile range, 0.75–0.92) while Pt2 and Pt1 were best described by the “existing genotype” distribution (0.00–0.51) (Figure 3*D*).

Next, we investigated the amino acid composition in the 3 novel variants at positions that have been associated with resistance to DAAs in well-characterized epidemic subtypes common in the Western world. In the NS3 gene of Pt1 and Pt2, we observed amino acid substitutions—36L, 80K, and 168E—that are associated with resistance in subtypes 1a, 1b, 2b, 3a, and 4d (Table 2). Of note, 36L is wild type in all subtypes except 1a, and 80K is wild type in the 4 subtype 8a reported to date and that were all successfully treated with DAAs [12]. Similarly, in the NS5A gene, amino acid substitutions 28V, 30S, 92S, and 93S, associated with resistance in 1a, 1b, 2b, and 4d, were detected in all 3 patients. Of note, 30S, 92S, and 93S are also wild type in subtype 8a. In contrast, a single amino acid substitution associated with resistance, 159F, was observed in the NS5B gene in Pt1.

**Table 2.** Analysis of Resistance-Associated Polymorphisms in Novel Variants^a^

Sequence	NS3			NS5A								NS5B		
	36	80	168	24	28	30	31	58	62	92	93	159	237	316
1a	V	Q	D	K	M	Q	L	H	E	A	Y	L	E	C
1b	L	Q	D	Q	L	R	L	P	Q	A	Y	L	E	N
2b	L	G	D	S	L	K	M	P	N	C	Y	L	E	C
3a	L	Q	Q	S	M	A	L	P	A	E	Y	L	E	C
4d	L	Q	D	K	L	R	M	P	E	A	Y	L	E	C
Consensus^b^	V/L	Q/G	D/Q	K/Q/S	M/L	Q/R/K/A	L/M	H/P	E/Q/N/A	A/C/E	Y	L	E	C/N
8a	L	K	D	K	L	S	L	P	V	S	S	L	L	C
Patient 1	L	*L*	**E^c^**	K	M	**S^d^**	L	P	A	A	*T*	**F^e^**	*T*	C
Patient 2	L	**K^f^**	D	K	*F*	**S^d^**	L	P	**L^g^**	**S^h^**	**S^i^**	L	*T*	C
Patient 3	L	Q	D	K	**V^j^**	A	L	P	A	A	**S^i^**	L	E	C

^a^Resistance-associated substitutions (RASs) occurring in patients 1, 2, and 3 are shown in boldface and defined as substitutions at major amino acid positions associated with reduced susceptibility in subtypes 1a, 1b, 2b, 3a, and 4d to direct-acting antivirals (DAAs) commonly used for treating nonepidemic subtypes (glecaprevir, voxilaprevir, daclatasvir, ledipasvir, pibrentasvir, velpatasvir, and sofosbuvir). Italic entries represent substitutions at major amino acid positions that are not wild type in epidemic subtypes but have not been shown to be associated with resistance to DAAs. RAS sites that are conserved are not shown (NS3: Y56, R155, and A156; NS5A: P32; NS5B: S282 and V321).

^b^Consensus wild-type residues in epidemic subtypes.

^c^In NS3, 168E confers resistance to glecaprevir (subtype 2b) and voxilaprevir (subtype 4d).

^d^In NS5A, 30S confers resistance to daclatasvir, ledipasvir (subtype 4d), and velpatasvir (subtype 1b).

^e^In NS5B, 159F confers resistance to sofosbuvir (subtypes 1a, 1b, 2b, and 3a).

^f^In NS3, 80K confers resistance to glecaprevir (subtype 3a) and voxilaprevir (subtypes 1a and 3a).

^g^In NS5A, 62L confers resistance to daclatasvir (subtype 3a).

^h^In NS5A, 92S confers resistance to velpatasvir (subtype 2b).

^i^In NS5A, 93S confers resistance to daclatasvir (subtype 1a), ledipasvir (subtypes 1a and 4d), and velpatasvir (subtype 1a, 1b, 4d, and genotype 6).

^j^In NS5A, 28V confers resistance to daclatasvir, ledipasvir (subtypes 1a and 4d), and velpatasvir (subtype 1a and genotype 6).

Despite the presence of the various RASs, all 3 individuals achieved SVR12 with pangenotypic DAAs, sofosbuvir-velpatasvir (Pt1 and Pt2), and glecaprevir-pibrentasvir (Pt3) (Table 3). All 3 individuals also had no significant liver fibrosis before initiation of therapy.

**Table 3.** Treatment Outcomes

Patient	DAA Regimen	Treatment Outcome	Disease Stage^a^	Age at Treatment Initiation, y
Patient 1	Sofosbuvir-velpatasvir (12 wk)	SVR12	No fibrosis	44
Patient 2	Sofosbuvir-velpatasvir (5 wk)^b^	SVR12	No fibrosis	55
Patient 3	Glecaprevir-pibrentasvir (8 wk)	SVR12	No fibrosis	39

Abbreviations: DAA, direct-acting antiviral; SVR12, sustained virological response at 12 weeks.

^a^Stage determined by transient elastography (Fibroscan).

^b^The 12-week course was stopped after 5 weeks due to an adverse reaction.

## DISCUSSION

In this report, we identified novel HCV variants in 3 individuals residing in the United Kingdom but born overseas. The first 2 variants (Pt1 and Pt2) are potentially new subtypes of the recently identified GT8. The 2 variants were identified in individuals born in Southern India and Pakistan, respectively. In contrast, the 4 subtype 8a full genomes identified to date were sampled from individuals born in the Punjab region of Northern India but residing in Canada [12]. Two additional potential cases of HCV subtype 8a infections have been described in a couple residing in Australia who were also originally from the Punjab region in India, but only partial genome data was generated for one of the viruses [17]. Thus, our findings confirm the endemicity of GT8 in the subcontinent of India and expand the diversity of this genotype. It is likely that more subtypes of GT8 are yet to be identified in this region, and there may be a potentially large number of individuals with GT8 infection. The latest estimates are that 7.4 million (95% uncertainty interval, 5.8–10.0 million) and 6.1 million (5.0–14.9 million) people are living with viremic HCV in Pakistan and India, respectively [31], and therefore the region is a key target for HCV elimination.

Our analyses show that the third novel variant is probably a new HCV genotype. Comparison of pairwise genetic distances of the variant against representative reference subtype sequences for GT1–GT8 showed that it was more similar to GT6; however, it was also close in distance to other genotypes across the majority of the genome. In addition, the overall genetic distance distribution of the coding sequence overlapped the arbitral intragenotype and intergenotype range (25%–30%). Phylogenetic reconstruction further supports this notion, as the Pt3 branch was located separately and almost equidistant between GT6 and GT8 clusters, with robust branch support. The close similarity to GT6 in the pairwise genetic distance analyses could be due to the lack of similar or closely related genotype sequences, as evidenced by the analyses of known subtypes where the reference data set had the relevant subtype or genotype reference sequences removed.

Pt3 was of Indian heritage but born in Guyana, South America, suggesting that this genotype may be endemic in either or both regions. Guyana is culturally considered to be part of the Caribbean region, where GT1 has been estimated to be the most prevalent at 83%, followed by GT2 at 7% [32]. It has a significant Indian population (approximately 40%) who trace their ancestry to the Indian subcontinent (mostly from Uttar Pradesh, Bihar, and Jharkhand; Figure 2*A*) and immigrated as early as the beginning of the 19th century as servants and settlers [33, 34]. Most of the genotyping data in the Caribbean has been generated using commercial molecular assays; therefore, further characterization of circulating HCV variants in this region using WGS is warranted [35].

All 3 novel variants were mistyped by commercial or in-house molecular assays, including LiPA and partial NS5B genome sequencing. Similarly, all 6 cases of subtype 8a identified to date had their virus typed as GT5 by commercial LiPA or real-time PCR assays [7, 12], a similar result to that obtained for Pt1 in the current study. Using the WGS data, all 3 novel variants were identified as HCV by well-established and publicly available genotyping tools, HCV-GLUE [36] and Geno2pheno[hcv] [30], but both tools failed to conclusively assign a genotype or subtype to all 3. The HCV-GLUE software identifies HCV subtype by phylogenetic reconstruction, and Geno2pheno[hcv] uses a similar method. At the time of analysis, each software used a set of well-characterized subtype reference sequences consisting of GT1–GT7 but not GT8.

Of interest, the generation of full-length genomes of these novel variants that are 20%–30% genetically different from the probe set used for enrichment in our sequence capture assay validates the robustness and agnosticity of this approach for identifying novel variants. The genome from Pt1 had the p7-NS2 region missing, which was filled by a target-specific PCR amplicon approach. Subgenomic deletions have been described before in several HCV-infected individuals and are thought to be capable of autonomous replication, presumably by co-opting missing gene products from coinfecting wild-type virus present at low frequencies [37–40]. However, no short reads were recovered spanning the missing region from Pt1 next-generation sequencing data. We therefore cannot rule out the possibility that the missing region was a result of missing data or that the recovered p7-NS2 subgenomic segment was not from a coinfecting virus.

The diversity exhibited by HCV has consequences with regard to antiviral treatment outcomes. The legacy interferon-based therapies were more successful against GT2 and GT3 but less effective against GT1 [41]. On the other hand, first-generation protease inhibitors were most effective against subtypes 1a and 1b, whereas second- and third-generation DAAs, the latter considered pangenotypic, are highly efficacious against a broad range of genotypes with success rates >95%. However, recent evidence shows that some HCV subtypes that are rarer in the Western world, but endemic in low- and middle-income countries, are inherently resistant to DAAs, including some pangenotypic regimens, and exhibit significantly lower SVR12 rates. These include subtypes 1l, 3b, and 4r, which are endemic in West Africa, South Asia, and Central Africa, respectively [4–9]. They have been associated with the presence of polymorphisms, especially in the NS5A gene, which are linked with DAA resistance in epidemic subtypes (eg, 1a and 3a). Interestingly, all 3 novel variants identified in this study were successfully treated with pangenotypic regimens, despite the presence of RASs at several sites in NS5A gene and a couple in NS3 and NS5B, especially in Pt1 and Pt2.

Considering the current and previous reports of treatment outcomes for HCV GT8, most individuals (5 of 7) received second-generation, pangenotypic DAAs (sofosbuvir-velpatasvir in 3 and sofosbuvir-velpatasvir-voxilaprevir in 2), while 1 received sofosbuvir-daclatasvir or sofosbuvir-ledipasvir-ribavirin [12, 17]. SVR12 was achieved in all. Infection resolved spontaneously in the eighth individual. Other factors in addition to viral genotype, such as liver disease stage, have been shown to play a role in DAA therapy outcomes [42]. Of note, all 3 individuals in the current study had no detectable liver fibrosis. Liver disease stage information was available for 4 of the 6 patients with subtype 8a, with 3 having minimal liver fibrosis and 1 being cirrhotic. Further data on optimal treatment regimens in this genotype are required.

Our findings further expand the diversity of HCV. It has been hypothesized that the broad diversity of HCV and the associated geographic distribution and endemicity suggest that this may be a result of multiple cross-species transmissions followed by some virus-host codivergence. However, the discovery of potential animal virus homologues or reservoirs for HCV has proved elusive. Alternatively, it is possible that this could be a result of a single cross-species jump that occurred thousands of years ago, followed by virus-host codivergence in humans over a very long period [43].

In conclusion, our report describes 2 new subtypes belonging to GT8 (provisional subtypes 8b and 8c) and potentially a new GT9. The latter meets the ICTV mandate that 1 full-length complete coding sequence is required to confirm a new HCV genotype but ≥3 epidemiologically unlinked isolates with ≥1 having a complete coding region are required to assign a new subtype. The successful treatment of all 3 novel variants and the previous subtype 8a cases engenders confidence in the prospect that the strategic goal to eliminate HCV globally by 2030 can be achieved if the new DAAs are rolled out in regions where these novel variants are endemic. However, continued public health surveillance is required to discover additional cases belonging to these new subtypes and to detect the emergence or circulation of variants that may be refractory to DAA therapy.

## Supplementary Data

Supplementary materials are available at *The Journal of Infectious Diseases* online (http://jid.oxfordjournals.org/). Supplementary materials consist of data provided by the author that are published to benefit the reader. The posted materials are not copyedited. The contents of all supplementary data are the sole responsibility of the authors. Questions or messages regarding errors should be addressed to the author.

## Supplementary Material

jiae253_Supplementary_Data

## Notes

***Acknowledgments.*** We acknowledge members of the National Institute for Health and Care Research Health Protection Research Unit in the Blood Borne and Sexually Transmitted Infections (BBSTI) Steering Committee: Caroline Sabin (Health Protection Research Unit director), John Saunders (UK Health Security Agency lead), Catherine Mercer, Hamish Mohammed, Greta Rait, Ruth Simmons, William Rosenberg, Tamyo Mbisa, Rosalind Raine, Sema Mandal, Rosamund Yu, Samreen Ijaz, Fabiana Lorencatto, Rachel Hunter, Kirsty Foster, and Mamoona Tahir.

***Disclaimer.*** The views expressed are those of the authors and not necessarily those of the National Institute for Health Research, the UK Health Security Agency, or the Department of Health and Social Care.

***Financial support*.** This work was supported by the National Institute for Health and Care Research Health Protection Research Unit (NIHR HPRU) in Blood Borne and Sexually Transmitted Infections at University College, London, in partnership with the UK Health Security Agency; the National Institutes of Health (grant R01AI087520 to T. L.); and the Los Alamos National Laboratory (Laboratory Directed Research and Development program fellowship to Z. L. [project no. 20230873PRD4]).
