# Supplementary material for: Identification of 2 Novel Subtypes of Hepatitis C Virus Genotype 8 and a Potential New Genotype Successfully Treated With Direct Acting Antivirals
Source: J Infect Dis. 2024 May 8;230(6):e1254–62. doi: 10.1093/infdis/jiae253 (PMC11646602; doi:10.1093/infdis/jiae253)
Supplement: jiae253_Supplementary_Data [file jiae253_supplementary_data.docx]

**Figure S1: Proportion overlap of pairwise genetic distance between genotypes.** (A) For each window (x-axis) of length 500 with a step size of 50, the proportion of overlap (y-axis) between the pairwise distance distributions (kernel density estimations) of the patient’s virus sequence and the genotype most closely related versus the patient’s virus sequence and all other genotypes. The grey line indicates the cutoff given the established reference sequences for each subtype and genotype. Above the grey line indicates an uncertain genotype classification for a given window. The HCV genes are depicted below the x-axis. (B) Identical plots to panel A except that Pt2 is included as a genotype 8 reference sequence.
